# Interspecies rice *versus Arabidopsis thaliana* protein–protein interactome profiling by touch‐down overlapping PCR coupled with HiFi long‐read sequencing

**DOI:** 10.1111/jipb.70107

**Published:** 2025-12-04

**Authors:** Jie Huang, Yu Cheng, Jing Ruan, Xixi Liu, Dandan Xia, Yiting Chen, Delong Fan, Jiezheng Ying, Yifeng Wang, Xiaohong Tong, Zhiyong Li, Dawei Xue, Jianwei Zhang, Jian Zhang, Yuxuan Hou

**Affiliations:** ^1^ State Key Lab of Rice Biology and Breeding China National Rice Research Institute Hangzhou 311400 China; ^2^ College of Life and Environmental Sciences Hangzhou Normal University Hangzhou 311121 China; ^3^ National Key Laboratory of Crop Genetic Improvement, Hubei Hongshan Laboratory Huazhong Agricultural University Wuhan 430070 China

## Abstract

Touch‐down overlapping PCR coupled with HiFi long‐read sequencing, a high‐throughput method for large‐scale profiling of protein‐protein interactions based on stitch‐PCR identified 7,726 high‐confidence interactions between rice and *Arabidopsis* proteins by integrating a library‐vs‐library yeast two‐hybrid strategy with optimized PCR and long‐read sequencing.

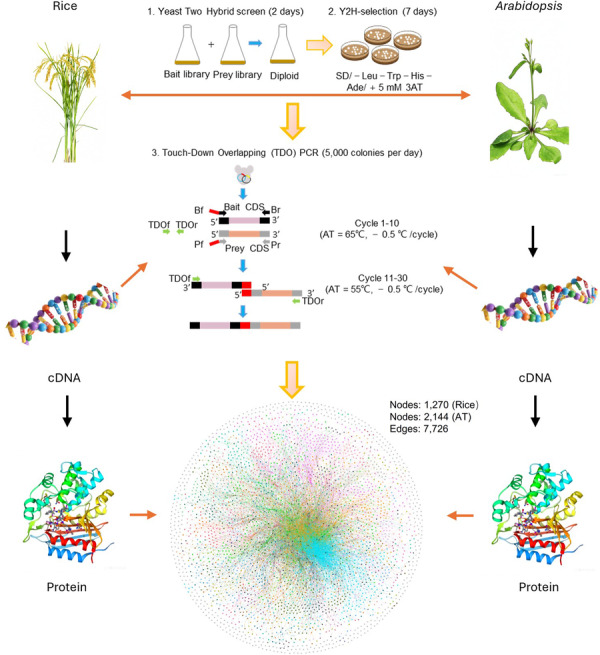

Protein–protein interactions (PPIs) occur when two or more proteins bind together, often resulting in the formation of complexes that perform essential functions within cells. PPIs are crucial for a variety of cellular activities, including signal transduction, metabolism, and immune response, which makes large‐scale identification of PPIs a primary focus of modern biological research (Ge et al., 2003). Several methodologies have been developed to map the PPIome, including homology‐based prediction methods and experimental techniques such as affinity purification mass spectrometry (AP‐MS) and yeast two‐hybrid (Y2H) assays. To scale up the PPI profiling by Y2H, several pipelines essentially fixed the bait‐prey association by integrating their cDNAs into one DNA fragment. For example, Stitch‐Seq and PLATE‐seq link the genes of bait and prey proteins through PCR amplification (Yu et al., 2011), while the CrY2H‐seq and RLL‐Y2H approaches utilize Cre recombinase‐mediated fusion of bait and prey cDNA fragments, thus enabling the simultaneous identification of thousands of bait‐prey linked fragments by next‐generation sequencing (Trigg et al., 2017; Yang et al., 2018). Recently, a barcode PCR‐based method known as BIP‐seq has been introduced, mapping 23,032 rice PPIs with a verification ratio exceeding 60% (Liu et al., 2025). However, its efficiency is limited by the requirement for a large number of barcode primers and laborious PCR procedures. Therefore, there is an urgent need for the development of scalable, cost‐effective, and precise methodologies to address the technical challenges. In this study, we present the development of touch‐down overlapping PCR coupled with HiFi long‐read sequence (TDOP‐seq), a massive and cost‐effective; Y2H‐based pipeline for profiling PPIomes. We demonstrated its capabilities by exploring the heterozygous PPIome between rice (*Oryza sativa*) and *Arabidopsis thaliana*, which could potentially enhance the characterization of rice proteins by leveraging functional insights from interactive *Arabidopsis* proteins.

TDOP‐seq is essentially built on the combination of library versus library Y2H mating strategy, touch‐down overlapping PCR on yeast colonies, and massive pac‐bio sequencing of PCR amplicon pools (Figure 1A). The bait library versus prey library mating strategy enables the obtaining of tens of thousands of random PPI colonies without large‐scale bait and prey ORFeomes construction. Utilizing PPI positive yeast colonies as templates, TDO‐PCR is performed in a single tube containing three pairs of primers (Figures 1A, S1A). The primer pairs Bf + Br and Pf + Pr are designed with high melting temperatures (Tm) to amplify the coding sequences (CDS) from the bait and prey vectors, respectively. A linker sequence of 29‐nt is attached to the 5' end of both Bf and Pf to ensure that the amplified bait and prey CDS share overlapping ends. The primers TDOf and TDOr are designed with lower Tm values that correspond to the 3’ ends of the bait and prey CDS, respectively. In the initial 10 thermal cycles, the annealing temperature is set to 65°C, allowing only the Bf + Br and Pf + Pr primers to function effectively for amplification. To improve the PCR amplification of the bait and prey CDS fragments, a touch‐down procedure is applied, decreasing the temperature by 0.5°C per cycle. Subsequently, 20 additional cycles are carried out at 55°C, also with −0.5°C/cycle. At this lower temperature, high dosage of TDOf + TDOr primers gains a competitive advantage, facilitating the overlapping PCR that stitches the bait and prey CDS sequences into a single fragment. Ultimately, tens of thousands of TDO‐PCR amplicons can be pooled for HiFi long‐read sequencing. It is noteworthy that the bait and prey cDNA sequences found within the same read originate from the same PPI‐positive yeast colony, thus could be classified as PPI pairs. Traditional overlapping PCR typically involves three rounds to combine the bait and prey cDNA into a single fragment, achieving an amplification rate of 95% ± 2.23%. In contrast, one‐step overlapping PCR employs primers with contrasted concentrations and Tm for conditional amplification across different annealing temperatures, significantly simplifying the process to complete the entire reaction in a single tube. Moreover, the implementation of a touch‐down procedure has markedly enhanced amplification efficiency, increasing it to over 75% compared to the 59% of the standard cycling procedure (Figure S1A, B).

**Figure 1:**
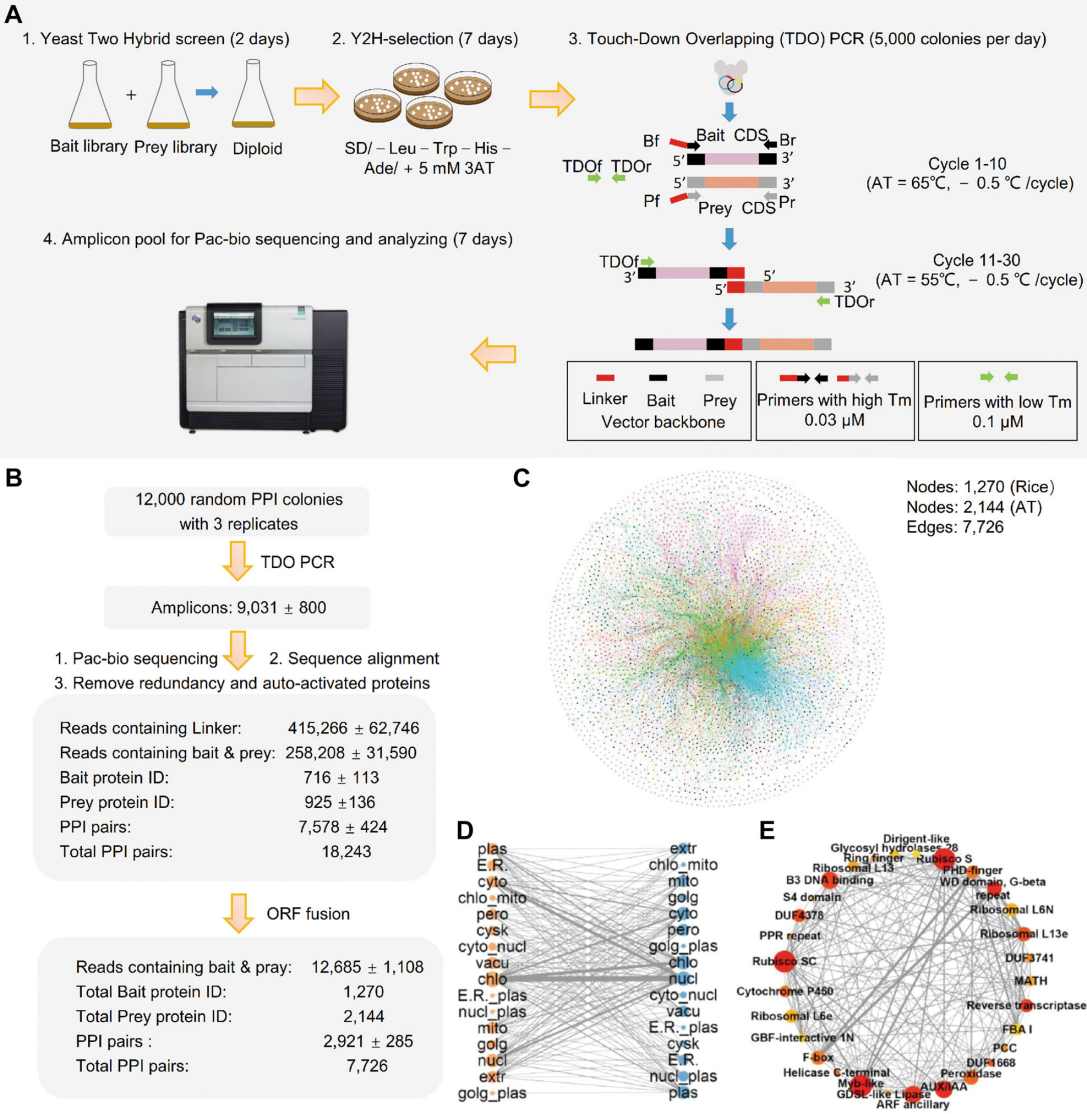
The workflow and bioinformatic analysis process of TDO‐PCR‐seq. **(A)** The workflow of TDOP‐seq technology. The procedure starts by crossing the bait and prey cDNA libraries in the Y2H system, which yields random PPI colonies on selective media. Subsequently, TDO‐PCR is performed in a single‐tube system using three primer pairs through a two‐stage amplification process. The first 10 high‐temperature cycles (starting at 65°C with a decrease of 0.5°C per cycle) preferentially amplify the coding sequences (CDS) of the bait and prey vectors, while the subsequent 20 low‐temperature cycles (starting at 55°C with a decrease of 0.5°C per cycle) utilize overlapping primers TDOf/TDOr to seamlessly splice the two CDS fragments into a complete segment. Finally, tens of thousands of the amplified bait and prey cDNA fragments are mixed and applied for HiFi sequencing. **(B)** The bioinformatic analysis pipeline and output of TDOP‐seq results on 36,000 random PPI colonies. **(C)** The overall picture of the identified PPIs between *Arabidopsis* and rice. The nodes represent proteins; the edges indicate PPI relationships. **(D)** Cellular localization interaction network. Nodes represent cellular components (Left: *Arabidopsis*; Right: rice), edges indicate interactions among proteins localized in the corresponding cellular compartments. **(E)** The map of interactions between conserved domains. Darker nodes indicate higher importance. Thicker edges represent stronger interactions. Larger nodes denote more interactions.

Currently, most of the reported PPIome profiling studies have been conducted within specific species; however, interspecies PPIome analysis holds significant importance in understanding host–pathogen interactions and in providing functional insights from well‐studied model organisms to others (Gordon et al., 2020). *Arabidopsis thaliana* and rice are two of the most crucial model species in plant molecular biology. Despite their importance, the functions of many of their genes remain poorly understood, highlighting the need for further functional information. To this end, we performed the TDOP‐seq procedure on 12,000 rice versus *Arabidopsis thaliana* PPI colonies across three independent replicates (Figure 1B). After aligning the linkers and removing auto‐activated proteins and duplicates, each replicate generated an average of 7,578 ± 424 PPIs, which encompassed approximately 716 bait proteins and 925 prey proteins. We further refined the PPIs by focusing on proteins with Gal4‐fused ORFs and ultimately obtained 2,921 ± 285 high‐confidence PPI pairs from each replicate (Figure 1B). In total, a network of 7,726 unique PPIs containing 1,270 rice nodes and 2,144 *Arabidopsis* nodes was constructed (Figure 1C; Table S1). Twenty‐five PPIs were randomly selected for validation using dual luciferase complementation assays (DLCA). Luminescence signals were observed in 16 out of the 25 tested pairs, yielding a positive interaction rate of 64% (Figure S2; Table S2). By systematically analyzing the length gap between bait and prey sequences from over 8 million reads, we found that TDO‐PCR could effectively integrate the bait and prey cDNA with a length gap ranging from 0 bp (equal length) to over 5 kb. Notably, the PPIs with a length gap over 1,000 bp were less preferentially amplified, indicating that a large length gap might interfere with the PCR amplification and PPI identification (Figure S3).

Analysis of subcellular localization indicates that protein–protein interactions may occur both intra‐ and inter‐cellularly, resulting in a complex and sophisticated network of localization‐based connections (Figure 1D; Table S4). Regarding PPIs between protein families with conserved domains, we identified 997 types of inter‐family interactions with at least 10 occurrences. Notably, no apical meristem (NAM) proteins and B3 binding domain proteins emerged as the most prevalent (Figure 1E; Table S5). A trait ontology analysis of the identified PPIs revealed nine PPI modules that are associated with disease resistance, plant growth, flowering, and various biological processes (Figure S4; Table S6). Assuming that interacting proteins tend to exhibit similar biological functions, we investigated the potential roles of five rice protein genes based on the known functions of their *Arabidopsis* counterparts, including replication factor C 1 (AtRFC1), which is a replication factor C subunit 1, regulating growth and development (Liu et al., 2010), WRKY DNA‐binding protein 33 (AtWRKY33), controlling salt tolerance (Jiang and Deyholos, 2008), and catalase 2 (AtCAT2), involved in the immune response (Yuan et al., 2017). Indeed, overexpression of uncharacterized *Prolamin precursor 18* (*OsPRO18*), *salt tolerance 204* (*OsST204*), *dirigent 19* (*OsDIR19*), and *dirigent 22* (*OsDIR22*) resulted in shorter root length, enhanced salt tolerance, and increased bacterial blight resistance, showing similar roles as their *Arabidopsis* counterparts AtRFC1, AtWRKY33, and AtCAT2, respectively (Figures S5–S7). Though trehalose‐6‐phosphate phosphatase 1 (OsTPP1) has been implicated in seed germination and cold tolerance, the interactive protein homeobox protein 33 (AtHB33), a zinc finger‐homeodomian transcription factor was involved in regulating both abiotic stress and pathogen‐inducible pattern (Qu et al., 2011). As expected, overexpression and knockdown of *OsTPP1* significantly improved and decreased blast resistance, respectively (Figure S8). The above results demonstrate that heterozygous PPI identification is an effective strategy for conveying functional information from one species to another.

In conclusion, we have developed TDOP‐seq, a massive Y2H‐based pipeline for PPIome profiling, and showcased its power by identifying 7,726 high‐confidence PPIs between rice and *Arabidopsis* within a month. In terms of time, cost, and accuracy, TDOP‐seq surpasses the established techniques, such as CrY2H‐seq, stitch‐PCR, and BIP‐seq, while delivering comparable throughput (Table S7). Despite its high throughput and efficiency, the current iteration of the TDOP‐seq method has several limitations that warrant discussion. First, it inherits a ~64% Y2H false‐positive rate, necessitating independent validation. Second, the TDO‐PCR fusion efficiency is ~75%, potentially causing undersampling and bias. Finally, reliance on long‐read sequencing remains cost‐prohibitive for widespread use, though decreasing costs may alleviate this. The resulting PPI network provides valuable functional insights that can enhance functional genomic studies in both species. We propose that TDOP‐seq holds significant potential for large‐scale PPIome profiling within or across species. An interspecies PPIome strategy would be highly effective in deepening our understanding of their biological interactions or in bridging knowledge from model organisms to non‐model species, ultimately aiding in crop improvement strategies.

## CONFLICTS OF INTEREST

These authors declare no conflicts of interest.

## AUTHOR CONTRIBUTIONS

J.H., Y.C., and J.R. contributed equally to this work. J.Z. and Y.H. planned and designed the research; J.H., J.R., X.L., Y.T.C., D.F., J.Y., Y.W., X.T., and Z.L. performed experiments; Y.C., J.W.Z., and D.X. analyzed the data; J.Z., Y.H., and J.H. wrote the manuscript. All authors read and approved this manuscript.

## Supporting information

Additional Supporting Information may be found online in the supporting information tab for this article: http://onlinelibrary.wiley.com/doi/10.1111/jipb.70107/suppinfo

Figure S1. (A) Comparison of PCR systems and procedures for the optimization of TDOP‐seq. (B) Agrose electrophoresis analysis on the PCR products.
Figure S2. Verification of 25 randomly selected PPIs in PPI dataset using DLCA.
Figure S3. Statistics on the density of interaction combinations of different length variations in CDS between bait and prey.
Figure S4. Nine functional modules of the identified PPIs based on trait ontology.
Figure S5. The genetic validation of OsPRO18 in regulating root development.
Figure S6. The genetic validation of OsST204 in regulating salt stress response.
Figure S7. The genetic validation of OsDIR19 and OsDIR22 in regulating rice disease resistance.
Figure S8. The genetic validation of OsTPP1 in regulating rice disease resistance.
Table S1. The 7,726 PPIs identified in this study.
Table S2. LUC verification of PPIs.
Table S3. Sequences of primers used in this study.
Table S4. Cellular localization of the detected PPI proteins.
Table S5. Conserved domain of the detected PPI proteins.
Table S6. Trait ontology analysis of identified PPIs.
Table S7. Comparison of the reported PPIome profiling methods

jipb‐2025‐0951‐File004

jipb‐2025‐0951‐File005

jipb‐2025‐0951‐File006

jipb‐2025‐0951‐File007

jipb‐2025‐0951‐File008

jipb‐2025‐0951‐File009
