# Supplementary material for: Interspecies rice versus Arabidopsis thaliana protein–protein interactome profiling by touch‐down overlapping PCR coupled with HiFi long‐read sequencing
Source: J Integr Plant Biol. 2025 Dec 4;68(2):294–6. doi: 10.1111/jipb.70107 (PMC12863018; doi:10.1111/jipb.70107)
Supplement: Supplementary file 1 — Figure S1. (A) Comparison of PCR systems and procedures for the optimization of TDOP‐seq. (B) Agrose electrophoresis analysis on the PCR products. Figure S2. Verification of 25 randomly selected PPIs in PPI dataset using DLCA. Figure S3. Statistics on the density of interaction combinations of different length variations in CDS between bait and prey. Figure S4. Nine functional modules of the identified PPIs based on trait ontology. Figure S5. The genetic validation of OsPRO18 in regulating root development. Figure S6. The genetic validation of OsST204 in regulating salt stress response. Figure S7. The genetic validation of OsDIR19 and OsDIR22 in regulating rice disease resistance. Figure S8. The genetic validation of OsTPP1 in regulating rice disease resistance. Table S1. The 7,726 PPIs identified in this study. Table S2. LUC verification of PPIs. Table S3. Sequences of primers used in this study. Table S4. Cellular localization of the detected PPI proteins. Table S5. Conserved domain of the detected PPI proteins. Table S6. Trait ontology analysis of identified PPIs. Table S7. Comparison of the reported PPIome profiling methods [file JIPB-68-294-s001.docx]

**Supplementary Materials and methods**

**Rice and** ***Arabidopsis* cDNA library construction and library vs library Y2H mating**

The normalized bait and prey Y2H cDNA libraries were established through a commercial service by Takara Bio Inc (Dalian, China). Briefly, mRNA of rice and *Arabidopsis* was isolated from the ten tissues (root, stem, leaf, panicle/flower, callus, seedling, and seed at 0, 3, 7, 15 DAP) and reverse transcribed using SMART cDNA library construction kit (Cat No. 634901, Clontech, Dalian, China), and directionally cloned into pGBKT7 and pGADT7 vectors, respectively, through the *SfiI* site. To detect positive Wise-pairs using high-throughput mating, we combined 0.8 mL each of the bait and prey cDNA library stocks (from Y187 and Y2HGold strains, respectively) in a sterile 2 L flask containing 50 mL of 2 × YPDA medium. The mixture was then incubated at 30°C for 20 hours with gentle shaking at 50 rpm. The mated zygotes were harvested and resuspended in 15 mL of 0.9% NaCl solution. They were then plated on selective SD/−Leu−Trp−His−Ade medium supplemented with 5 mM 3-AT and incubated at 30°C for 7 days. Yeast colonies exceeding 1-2 mm in diameter were scored as positive for protein-protein interactions (PPIs).

**High-throughput** **TDO-PCR amplification**

Individual yeast colonies exhibiting PPIs were either manually picked or collected using a QPix 420 high-throughput colony picker (Molecular Devices, San Jose, USA) and transferred into a 96-well plate containing 10 μL of freshly prepared 1× KBB lysis buffer (20 mM KOH, 0.5% BSA, 1 M betaine). Heat treated at 100℃ for 5 minutes. For the TDO-PCR, a 20 μL reaction system containing 10 μL 2 × rTaq Mix (Cat No. 21502-04, Tolobio, Shanghai, China), 7.8 μL H_2_O, 0.2 μL 0.33 μM TDO-AD-F/R and TDO-BD-F/R, 0.2 μL 10 μM TDO-AD-2R and TDO-BD-2R, and 1 μL yeast lysate in KBB lysis buffer were applied for PCR. The PCR procedure is shown in Figure S2. Finally, sixties of thousands of PCR products were collected, column purified, and quantified by Qubit Fluorometer (Novogene, Beijing，China) for HiFi long-read sequencing. The sequence of the primers can be found in Table S3.

**HiFi long-read sequencing and sequence demultiplex**

HiFi long-read sequencing was performed by Novogene ltd, Beijing, China. Briefly, PCR products were repaired for Blunt-End-Ligation using Template Prep Kit 1.0 - SPv3 (Cat No. 100-222-300, Pacific Biosciences, California, U.S.A.) and filtered for DNA >15 kb in size using Diagenode Megaruptor system (Cat No. B06010003, Diagenode, Belgium). Then, the filtered DNA was purified to produce the SMRTbell templates using AMPure PB Beads (Cat No. 102-182-500, Pacific Biosciences, California, U.S.A.), and treated with ExoⅢ (Cat No. M0206V, NEB, Beijing, China) and ExoⅦ (Cat No. M0379S, NEB, Beijing, China) to remove single strand DNA and DNA residues at 37 ℃ for 1 h. After DNA purification and concentration by AMPure PB Beads and quantification by Agilent DNA 12000 Kits (Cat No. 5067-1508RUO, Agilent, Beijing, China), sequencing barcodes were attached to the ends of the SMRTbell templates using polymerase from Binding Kit. Finally, the library was sequenced in SMRT Cells (Cat No. 102-202-200, Pacific Biosciences, California, U.S.A.) using DNA Sequencing Reagent Kit (Cat No. 101-597-900, Pacific Biosciences, California, U.S.A.).

The raw sequencing data were processed into circular consensus sequences (CCS) using pbccs (v6.4.0) software with parameters “--minPasses 3 --min-rq 0.99.” Next, the linker sequence was aligned to CCS reads in FASTA format (maximum mismatch and gap: 3 bp). CCS reads that matched multiple linker sequences were discarded. CCS reads containing linker sequence were then split into two parts, corresponding to bait and prey, respectively. Subsequently, the backbone sequences of pGADT7 and pGBKT7 vectors were aligned against the split CCS reads to determine their origin (maximum mismatch and gap: 2 bp). Similarly, split CCS reads containing backbone sequences from both the pGADT7 and pGBKT7 vectors were discarded. Following this, unpaired split CCS reads were also discarded, and the vector universal primer sequences (Bf+Br, Pf+Pr) as well as any flanking sequences beyond them were removed. To determine the ORF fusion with GAL4 domain, the backbone sequences flanking the original ORF initiation sites were removed from the qualiﬁed split CCS reads (maximum mismatch and gap: 2 bp). Then the clean reads of bait and prey were aligned to the CDS sequences of *Oryza sativa* L. ssp. Japonica (Nipponbare, Rice Genome Annotation Project Release 7, <http://rice.uga.edu/>) and *Arabidopsis thaliana* (Araport11, https://www.arabidopsis.org/) respectively, using BLASTN (v2.15.0) with parameters “-task blastn-short -outfmt 6 -evalue 1e-5”, to verify in-frame fusion and identify the gene IDs of proteins with the highest score. The two proteins derived from a single CCS read constituted one protein–protein interaction pair. Only pairs in which both protein sequences were fused in-frame were retained.

**Bioinformatics and statistical analysis**

The protein-protein interaction network was constructed and analyzed using Cytoscape (v3.10.3) and Gephi (v0.10.1). Network Analyzer and cytoHubba plugins in Cytoscape were employed to compute network parameters and identify hub genes. Protein domains of all interacting genes were obtained from InterProScan (v5.75-106.0). Trait ontology (TO) annotation utilized the Ontology Enrichment Analysis Tool (https://planteome.org/oat/). WoLF PSORT ([https://wolfpsort.hgc.jp](https://wolfpsort.hgc.jp/)) was employed to predict subcellular localization, and iTAK (v1.7) was used to predict transcription factors, transcription regulators and protein kinases. All experimental data are presented as mean ± SD, with statistically significant differences (α = 0.05) among groups determined by one-way ANOVA followed by Tukey's post hoc test for normally distributed datasets with equal variances, using GraphPad Prism 9 for all statistical analyses.

**Dual luciferase complementation assays**

To ensure an unbiased assessment of the overall false positive rate of our method, we employed an objective computational sampling approach. Specifically, we used a random sampling function in Microsoft Excel (=TAKE(SORTBY(A1:A7726, RANDARRAY(ROWS(A1:A7726))), 25)) to randomly select 25 unique bait-prey pairs from the total pool of candidates. The dual luciferase complementation assays (DLCA) were performed as previously described (Chen et al., 2008). The cDNAs of *Arabidopsis* and rice genes in pairs were inserted into pCAMBIA 1300-NLuc and pCAMBIA 1300-CLuc to construct the A-NLuc, R-CLuc vectors, respectively. The primers for vector construction are listed in Table S3. These binary vectors were transformed into *Agrobacterium tumefaciens* (strain EHA105). Bacterial suspensions were infiltrated into 6-week-old *N. benthamiana* leaves using needleless syringes. Then the tobaccos were cultured for 16-48 hours after injection. One millimolar luciferin (Promega) was sprayed onto leaves, and then the leaves were kept in the dark for 6 min to quench the fluorescence. The LUC image was observed with a low-light-cooled CCD imaging apparatus (NightShade LB 985; Berthold).

**Generation of transgenic plants**

The overexpression materials for *OsPRO18*, *OsST402,* *OsDIR19,* *OsDIR22 and* *OsTPP1* were generated as described below. The genes coding region was amplified with the gene-specific primers and cloned into vector pCAMBIA1300 (*Kpn*Ⅰ and *Pst*Ⅰ digested, *OsPRO18*, *OsST402*), pu1301 (*Kpn*Ⅰ and *Bam*HⅠ digested，*OsDIR19,* *OsDIR22*) and pCAMBIA1300 (*Kpn*Ⅰ and *Bam*HⅠ digested, *OsTPP1*), respectively. In which genes is driven by the cauliflower mosaic virus (CaMV) 35S promoter. The recombinant plasmids were transformed into Nipponbare variety by *Agrobacterium*-mediated method. Hygromycin-resistant calli were grown in artificial incubator to produce transgenic plants. T_0_ plants were grown in padding field of China National Rice Research Institute in Fuyang, Zhejiang and positive plants were confirmed by PCR and sequencing. The positive T_2_ plant were used for evaluation. The knockout (*ostpp1*) materials for *OsTPP1* were generated by Li et al. in our laboratory (Li et al., 2022). Primers for vector construction are listed in Table S3.

**Determination and analysis of rice seedling root and coleoptile length under dark cultivation**

Pre-germinated rice seeds were surface-sterilized, grown hydroponically in aerated nutrient solution (pH 5.5-6.0) under controlled conditions (28℃, dark) for 96 hours. After 4 days, seedlings were gently removed, and root length was measured either manually using a ruler (primary root + longest lateral roots) or digitally using ImageJ software. Data from 5 replicates (Each replicate consists of 10 seedlings) were recorded as mean ± standard deviation for analysis.

**Salt tolerance analysis**

The salt tolerance analysis was performed as previously described (Yu et al., 2023). The salt stress was imposed on the seedlings with two and one-half leaves by supplementing with 0 mM as control and 140 mM NaCl as salt treatment. The solutions were renewed every 2 days and the pH was maintained at 5-6. After 10 days of treatment, all plants were transferred to fresh Yoshida’s solution to recover for 10 days and then the survival rates (SR) were determined. In order to obtain accurate phenotypic data, three biological replicates were set up in the treatment group and the control group, and four plants were selected from each replicate for measurement. The criteria for selection were the four plants located in the block.

**Blast resistance test**

The virulent *M. oryzae* strain RB22 was used for inoculation assays. Six-week-old rice plants (*Oryza sativa* cv. Nipponbare) were punch-inoculated following established method (Park et al., 2012). A freshly prepared spore suspension (5.0 × 10⁵ spores/mL in 0.05% Tween-20) was applied (10 µL per site) to press-injured sites on fully expanded leaves. The leaves were then excised and disease progression was assessed by measuring lesion area at 6 days post-inoculation (dpi) using ImageJ.

**Blight resistance test**

The highly virulent *Xanthomonas oryzae* pv. *oryzae* (*Xoo*) strain LA20 (isolated from Anhui, China) was used for inoculation (Hou et al., 2023). At the booting stage, rice plants were inoculated using the leaf-clipping method. Briefly, LA20 was cultured to an OD600 of 0.5, and approximately 2 cm of leaf tips were cut with sterile scissors dipped in the bacterial suspension. Disease progression was evaluated at 14 days post-inoculation (dpi) by calculating the lesion length.

**Data availability**

The data used to support the ﬁndings of this study are available from the corresponding author upon request.

**References**

**Chen, H., Zou, Y., Shang, Y., Lin, H., Wang, Y., Cai, R., Tang, X., and Zhou, J.** (2008). Firefly luciferase complementation imaging assay for protein-protein interactions in plants. Plant Physiology **146**:323-324.

**Hou, Y., Liang, Y., Yang, C., Ji, Z., Zeng, Y., Li, G., and E. Z.** (2023). Complete genomic sequence of *Xanthomonas oryzae* pv. *oryzae* srain, LA20, for studying resurgence of rice bacterial blight in the Yangtze River region, China. International Journal of Molecular Sciences, **24**:8132.

**Li, Z., Wei, X., Tong, X., Zhao, J., Liu, X., Wang, H., Tang, L., Shu, Y., Li, G., Wang, Y., Ying, J., Jiao, G., Hu, H., Hu P., Zhang, J.** (2022). The OsNAC23-Tre6P-SnRK1a feed-forward loop regulates sugar homeostasis and grain yield in rice. Molecular Plant **15**:706-722.

**Park, C., Chen, S., Shirsekar, G., Zhou, B., Khang, C., Songkumarn, P., Afzal, A,, Ning, Y., Wang, R., Bellizzi, M., Valent, B., Wang, G.** (2012). The *Magnaporthe oryzae* effector AvrPiz-t targets the RING E3 ubiquitin ligase APIP6 to suppress pathogen-associated molecular pattern-triggered immunity in rice. Plant Cell. **24**:4748-62.

**Yu, J., Zhu, C., Xuan, W., An, H., Tian, Y., Wang, B., Chi, W., Chen, G., Ge, Y., Li, J., et al.** (2023). Genome-wide association studies identify OsWRKY53 as a key regulator of salt tolerance in rice. Nature Communications **14**:3350.


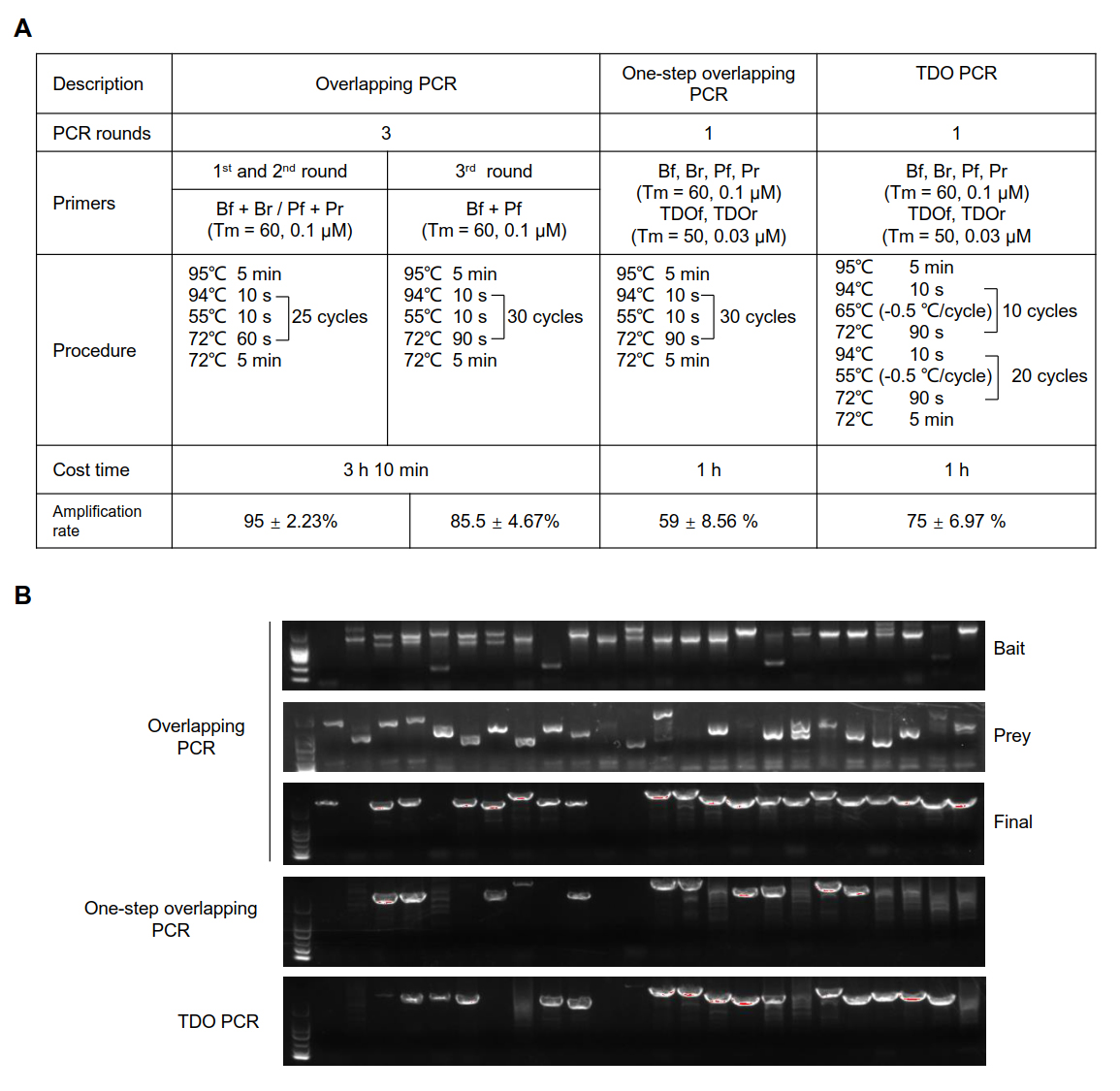
**Supplementary Figures**

**Figure S1.** **(A)** Comparison of PCR systems and procedures for the optimization of TDOP-seq. **(B)** Agrose electrophoresis analysis on the PCR products. The gel image shows the amplification products of the same 24 yeast monoclonal colonies. The primer sequences used in this experiment are provided in Supplementary Table 3.


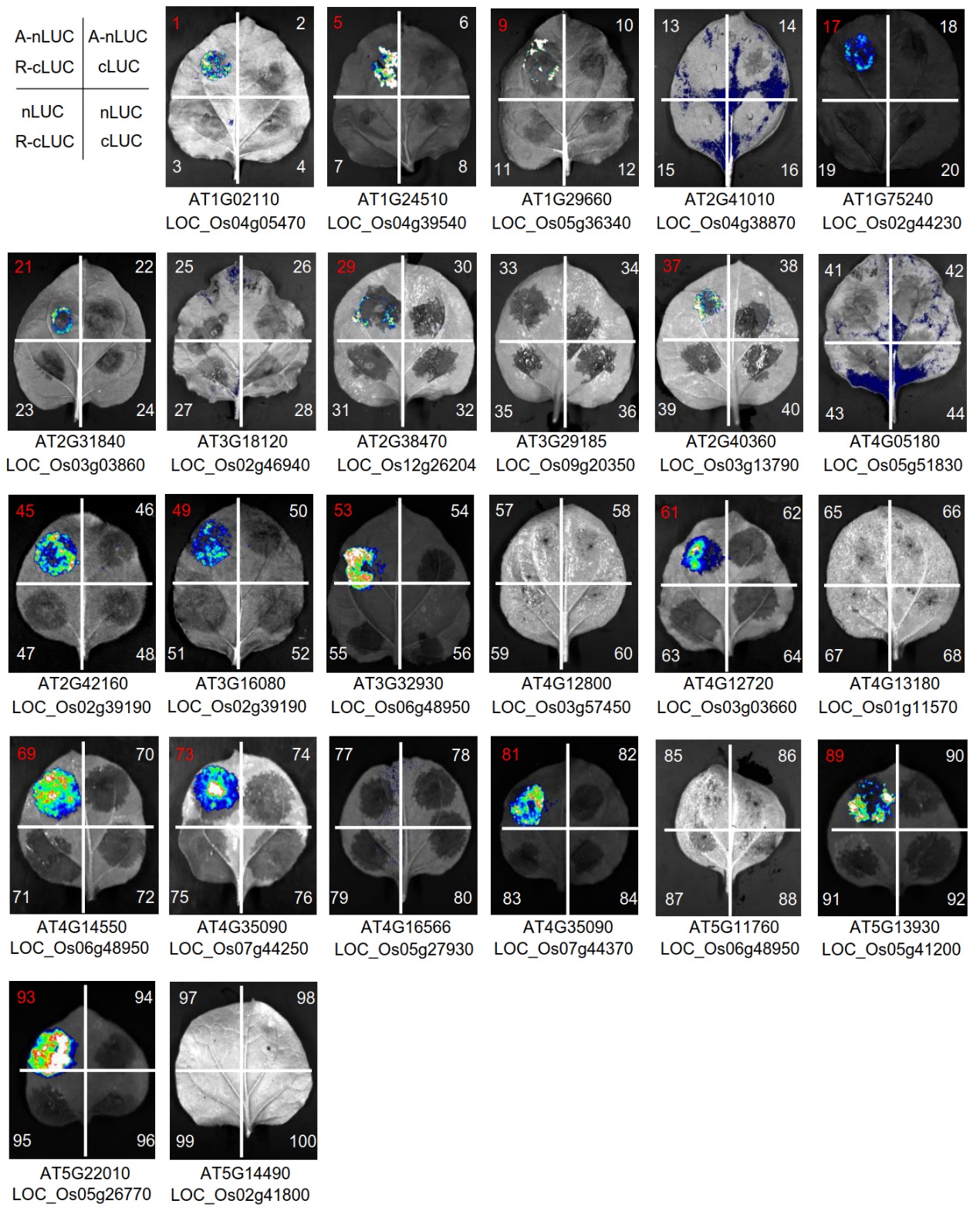


**Figure S2.** **Verification of 25 randomly selected PPIs in PPI dadaset using DLCA.** The red font indicates the positive PPI retested by DLCA. All the retested protein IDs can be found in Table S2


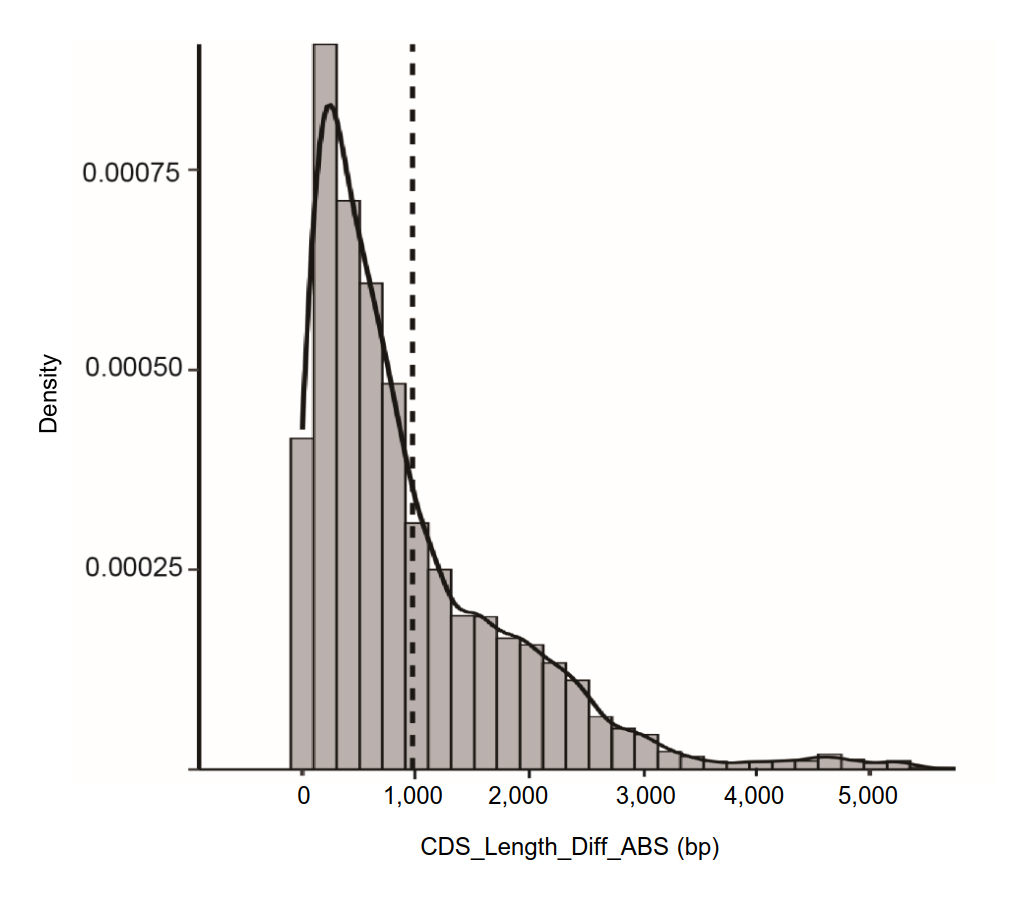


**Figure S3.** **Statistics on the density of interaction combinations of different length variations in CDS between bait and pray.** The horizontal axis represents the length difference between the CDS of Bait and Pray, in base pairs (bp). Grouped in 200 bp intervals, with the dashed line indicating the mean value.


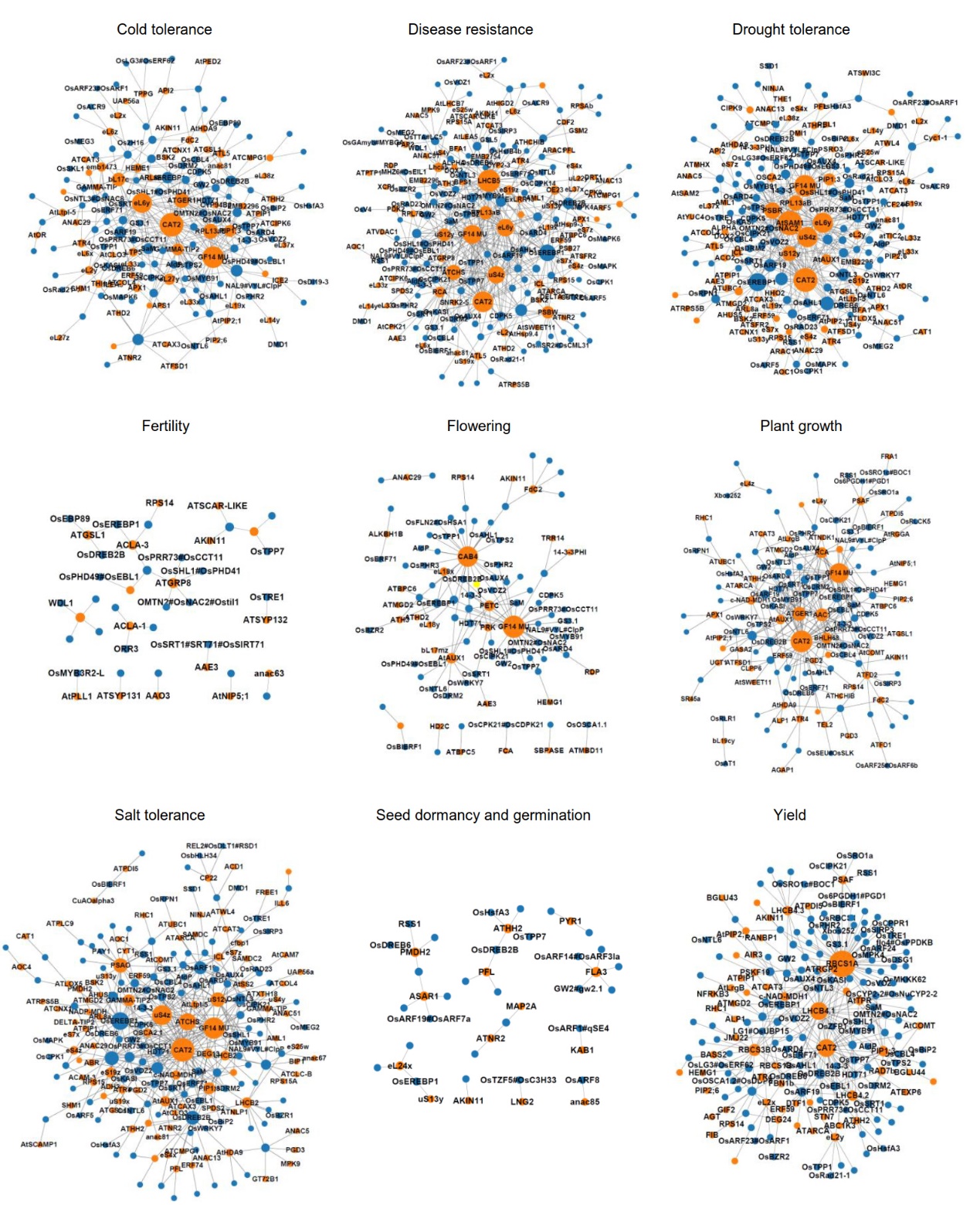


**Figure S4. Nine functional modules of the identified PPIs based on trait ontology.** The node size represents degree scores, with larger nodes representing greater scores and greater importance in the subnetwork. Orange nodes represent *Arabidopsis* proteins, blue nodes represent rice proteins.


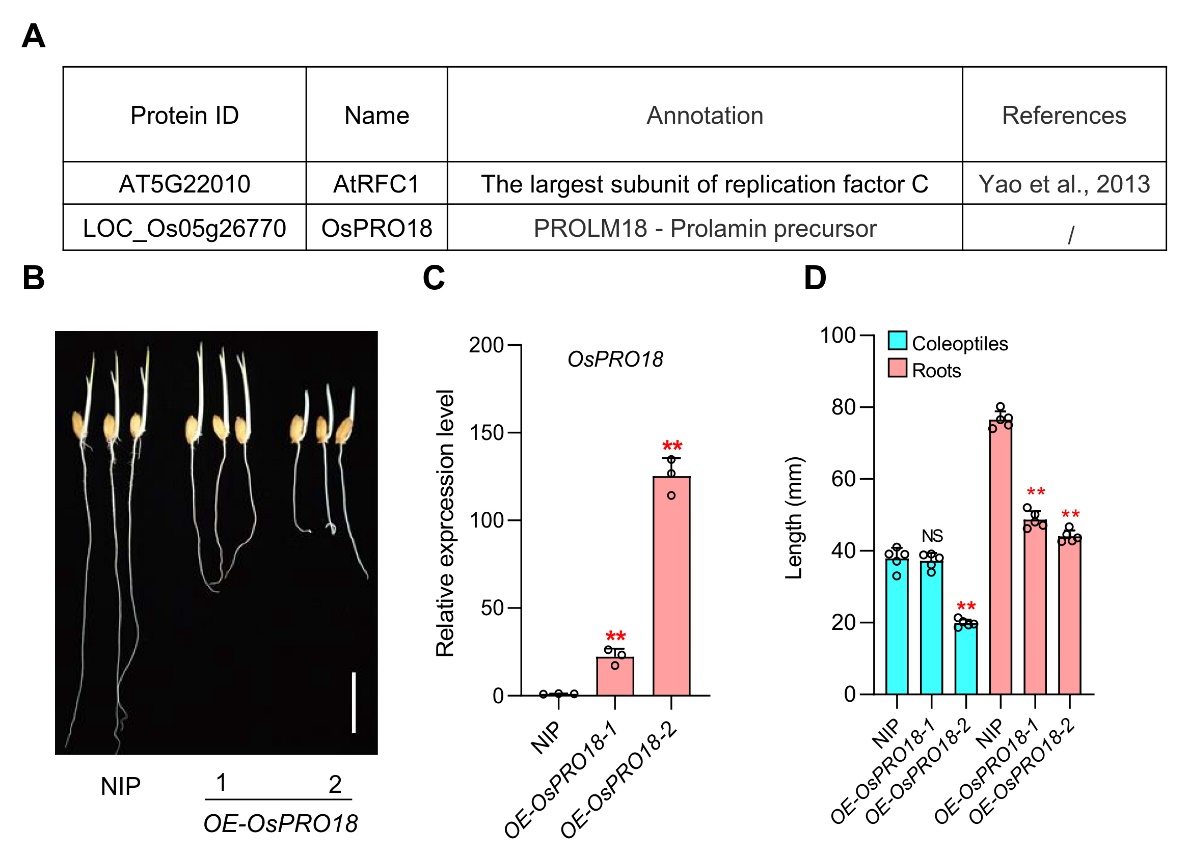


**Figure S5.** **The genetic validation of OsPRO18 in regulating root development. (A)** PPI information between OsPRO18 and AtRFC1. **(B)** The morphology of NIP and *OsPRO18* overexpression seedlings after 4 days of dark cultivation. Scale bar, 20 mm. **(C)** The relative transcriptional level of *OsPRO18* in NIP and overexpression transgenic plants. **(D)** The comparison of coleoptiles and root length of NIP and overexpression transgenic plants. The data is presented as mean ± *SD* (*n* = 5 plants), Tukey's test, ***P* < 0.01, ****P* < 0.001.


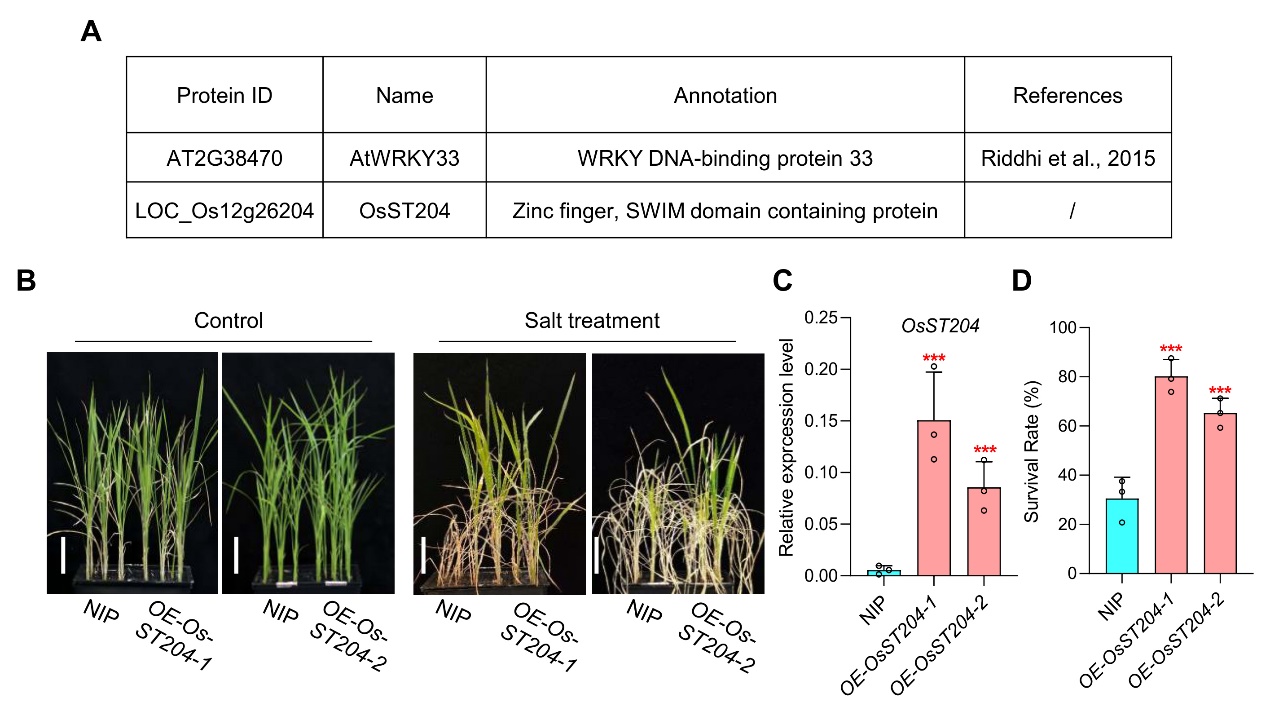


**Figure S6.** **The genetic validation of OsST204 in regulating salt stress response.** **(A)** PPI information between OsST204 and AtWRKY33. **(B)** Evaluation of salt tolerance of NIP, *OsST204* overexpression plants. Pictures were taken at 10 d post salt treatment, without (left) and with 200 mM NaCl (right). Scale bar, 5 cm. **(C)** The relative transcriptional level of *OsST204* in NIP and overexpression transgenic plants. **(D)** The comparison of survival rates in NIP and *OE-OsST204* transgenic plants under salt stress. The data is presented as mean ± *SD* (*n* = 3, Each replicate consists of 24 seedlings.), Tukey's test, ****P* <0.001.


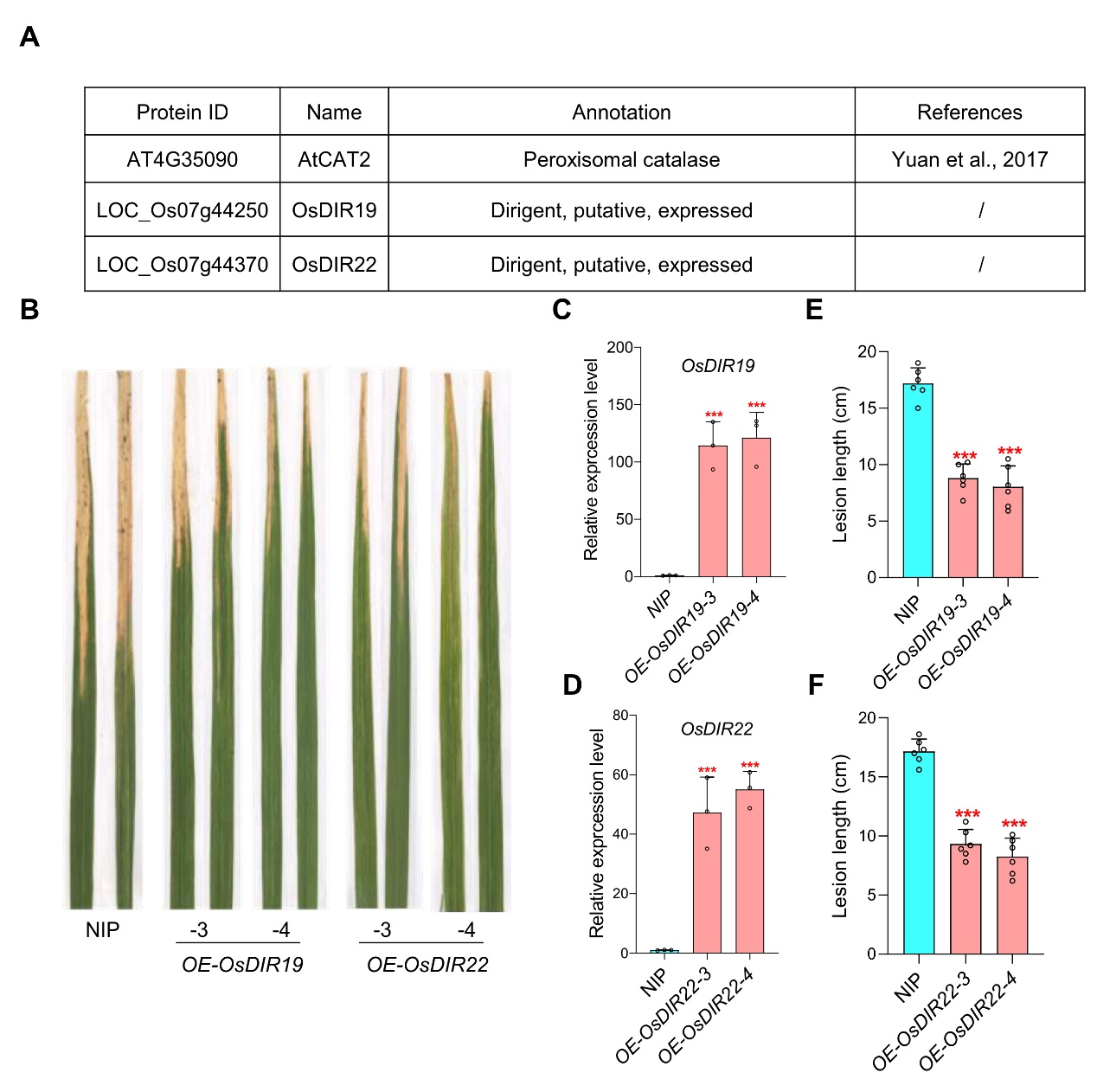


**Figure S7. The genetic validation of OsDIR19 and OsDIR22 in regulating rice disease resistance.** **(A)** PPI information among OsDIR19, OsDIR22 and AtCAT2. **(B)** Evaluation of bacterial blight resistance of NIP, *OsDIR19* and *OsDIR22* overexpression plants after 14 days of inoculation. **(C, D)** The relative transcriptional level of *OsDIR19* and *OsDIR22* in NIP and overexpression transgenic plants. **(E, F)** The comparison of lesion length of NIP and overexpression transgenic plants. The data is presented as mean ± *SD* (*n* = 6 plants), Tukey's test, ****P* < 0.001.


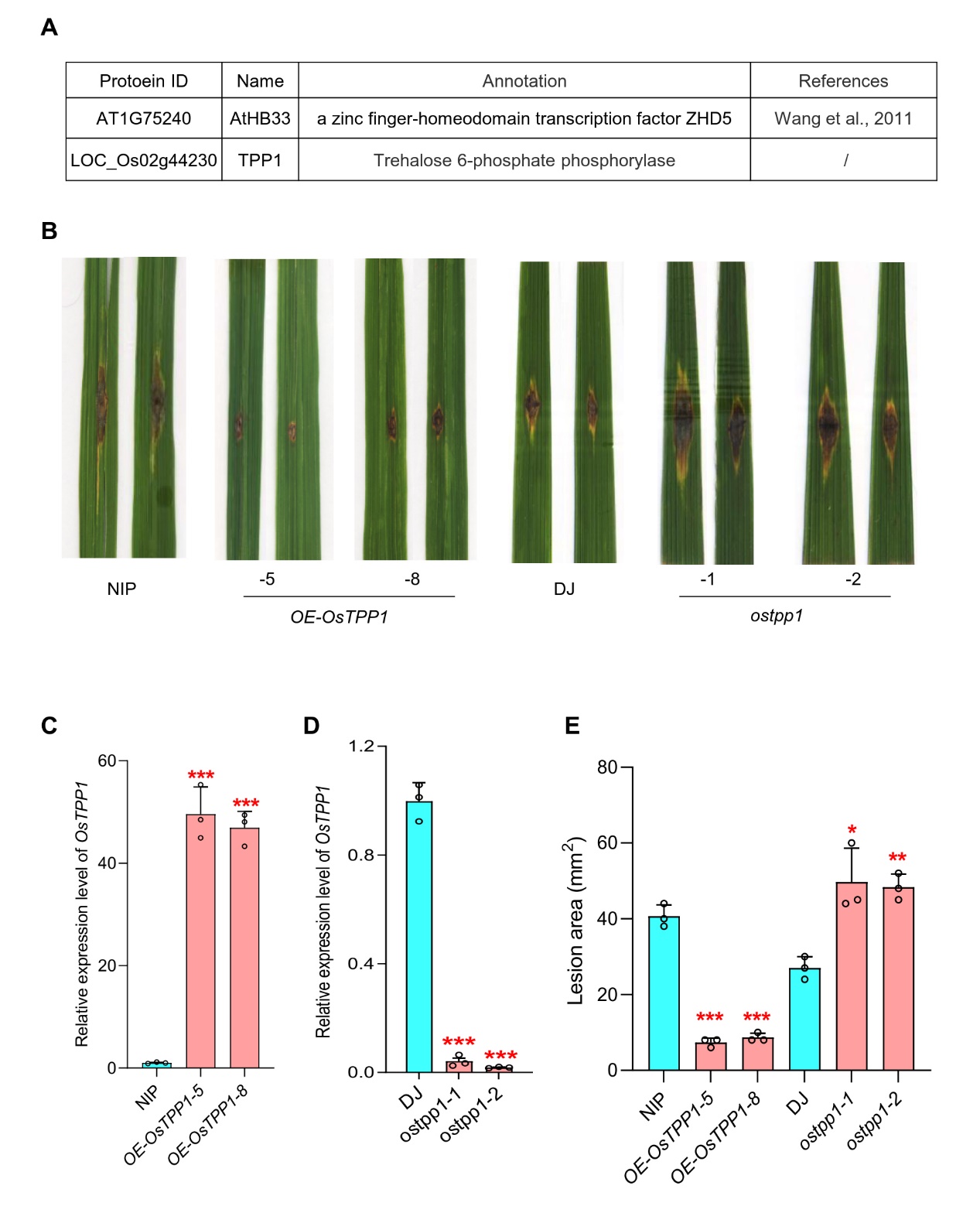


**Figure S8. The genetic validation of OsTPP1 in regulating rice disease resistance.** **(A)** PPI information between OsTPP1 and AtHB33. **(B)** Evaluation of blast resistance of NIP (WT), *OsTPP1* overexpression and Dongjing (DJ, WT), *ostpp1* plants after 6 days of inoculation. **(C)** The relative transcriptional level of *OsTPP1* in NIP and overexpression transgenic plants. **(D)** The relative transcriptional level of *OsTPP1* in DJ and *ostpp1* plants. **(E)** The comparison of lesion area in NIP and *OE-TPP1*, DJ and *ostpp1* plants. The data is presented as mean ± *SD* (*n* = 3), Tukey's test, **P* < 0.05, ***P* < 0.01, ****P* < 0.001.

**Supplementary tables**

**Table S1. The 7,726 PPIs identified in this study****.**

**Table S2. LUC verification of PPIs.**

**Table S3. Sequences of primers used in this study.**

**Table S4. Cellular localization of the detected PPI proteins.**

**Table S5. Conserved domain of the detected PPI proteins.**

**Table S6. Trait ontology analysis of identified PPIs.**

**Table S7. Comparison of the reported PPIome profiling methods.**

| **Supplementary Table 2. LUC verification of PPIs** | | | |
| --- | --- | --- | --- |
| **NO.** | **Prey protein ID** | **Bait protein ID** | **Interaction by LUC** |
| 1 | AT1G02110 | LOC_Os04g05470 | Positive |
| 5 | AT1G24510 | LOC_Os04g39540 | Positive |
| 9 | AT1G29660 | LOC_Os05g36340 | Positive |
| 13 | AT2G41010 | LOC_Os04g38870 | Negative |
| 17 | AT1G75240 | LOC_Os02g44230 (OsTPP1) | Positive |
| 21 | AT2G31840 | LOC_Os03g03860 | Positive |
| 25 | AT3G18120 | LOC_Os02g46940 | Negative |
| 29 | AT2G38470 | LOC_Os12g26204 (OsST204) | Positive |
| 33 | AT3G29185 | LOC_Os09g20350 | Negative |
| 37 | AT2G40360 | LOC_Os03g13790 | Positive |
| 41 | AT4G05180 | LOC_Os05g51830 | Negative |
| 45 | AT2G42160 | LOC_Os02g39190 | Positive |
| 49 | AT3G16080 | LOC_Os08g37920 | Positive |
| 53 | AT3G32930 | LOC_Os06g48950 | Positive |
| 57 | AT4G12800 | LOC_Os03g57450 | Negative |
| 61 | AT4G12720 | LOC_Os03g03660 | Positive |
| 65 | AT4G13180 | LOC_Os01g11570 | Negative |
| 69 | AT4G14550 | LOC_Os06g48950 | Positive |
| 73 | AT4G35090 | LOC_Os07g44250 (OsDIR19) | Positive |
| 77 | AT4G16566 | LOC_Os05g27930 | Negative |
| 81 | AT4G35090 | LOC_Os07g44370 (OsDIR22) | Positive |
| 85 | AT5G11760 | LOC_Os06g48950 | Negative |
| 89 | AT5G13930 | LOC_Os05g41200 | Positive |
| 93 | AT5G22010 | LOC_Os05g26770 (OsPRO18) | Positive |
| 97 | AT5G14490 | LOC_Os02g41800 | Negative |
